# Characterization and Engineering Studies of a New Endolysin from the *Propionibacterium acnes* Bacteriophage PAC1 for the Development of a Broad-Spectrum Artilysin with Altered Specificity

**DOI:** 10.3390/ijms24108523

**Published:** 2023-05-10

**Authors:** Christina Varotsou, Georgios E. Premetis, Nikolaos E. Labrou

**Affiliations:** Laboratory of Enzyme Technology, Department of Biotechnology, School of Applied Biology and Biotechnology, Agricultural University of Athens, 75 Iera Odos Street, 11855 Athens, Greece; cvarotsou@gmail.com (C.V.); giorgos.prem@gmail.com (G.E.P.)

**Keywords:** antimicrobial resistance, bacteriophage, endolysin, enzybiotics, peptidoglycan, Propionibacterium, inclusion bodies

## Abstract

The emergence of multidrug-resistant (MDR) bacteria has risen rapidly, leading to a great threat to global public health. A promising solution to this problem is the exploitation of phage endolysins. In the present study, a putative N-acetylmuramoyl-L-alanine type-2 amidase (NALAA-2, EC 3.5.1.28) from *Propionibacterium* bacteriophage PAC1 was characterized. The enzyme (*Pa*Ami1) was cloned into a T7 expression vector and expressed in *E. coli* BL21 cells. Kinetics analysis using turbidity reduction assays allowed the determination of the optimal conditions for lytic activity against a range of Gram-positive and negative human pathogens. The peptidoglycan degradation activity of *Pa*Ami1 was confirmed using isolated peptidoglycan from *P. acnes*. The antibacterial activity of *Pa*Ami1 was investigated using live *P. acnes* cells growing on agar plates. Two engineered variants of *Pa*Ami1 were designed by fusion to its N-terminus two short antimicrobial peptides (AMPs). One AMP was selected by searching the genomes of *Propionibacterium* bacteriophages using bioinformatics tools, whereas the other AMP sequence was selected from the antimicrobial peptide databases. Both engineered variants exhibited improved lytic activity towards *P. acnes* and the enterococci species *Enterococcus faecalis* and *Enterococcus faecium*. The results of the present study suggest that *Pa*Ami1 is a new antimicrobial agent and provide proof of concept that bacteriophage genomes are a rich source of AMP sequences that can be further exploited for designing novel or improved endolysins.

## 1. Introduction

Antimicrobial resistance (AMR) is one of the most serious global threats of this century. The continuous increase in the emergence of multidrug-resistant (MDR) bacteria is estimated to raise the number of deaths to 10 million annually by 2050 [1,2]. The exploitation of endolysins is a promising approach for the treatment of infections caused by MDR bacteria [3,4,5,6,7]. Endolysins are hydrolytic enzymes encoded by bacteriophages and degrade the peptidoglycan (PG) layer of the host bacterial cell at the end of the lytic replication cycle, causing disruption of the cell’s integrity. Treatment of bacteria with endolysins provides major advantages over the administration of antibiotics, including immediate bactericidal activity, high target specificity, low toxicity to mammalian cells, and a low to minimum risk of developing resistance [1,2,3,4,5,6,7,8,9,10,11,12,13,14,15,16,17].

The cell wall of the bacteria consists of the polysaccharide peptidoglycan (PG), which is composed of alternating N-acetylglucosamine (GlcNAc) and N-acetylmuramic acid (MurNAc) residues linked by β-1→4 bonds. The glycan strands are cross-linked via short peptides linked to each MurNAc residue [18,19,20]. The bacterial cell wall may also contain additional structural components, such as teichoic and lipoteichoic acids, as well as macromolecules like fatty acids, polysaccharides, and proteins. The exact composition of the cell wall depends on the bacterial strain and differs between Gram-negative, Gram-positive, and acid-fast bacteria [8,18,19,21,22,23].

Endolysins are classified into three major groups: amidases, peptidases, and glucosidases [19], based on the specific bond in the peptidoglycan layer of the host’s cell wall that they cleave. The structure of phage endolysins is different between those that cleave the PG of Gram-negative or positive bacteria as a consequence of the differences in the structure of their PG. Typically, endolysins that lyse Gram-positive bacteria consist of two distinct functional domains, namely the cell wall binding domain (CBD) and the enzymatically active domain (EAD). On the contrary, the enzymes that lyse Gram-negative bacteria are smaller and consist of a single EAD [3].

*Propionibacterium acnes* is an aerotolerant, anaerobic, Gram-positive bacterium that normally colonizes the sebaceous glands on human skin. It is characterized as a dominant human skin commensal. Over 80% of adolescents and an increasing number of adults are affected by acne vulgaris, a skin condition caused by *Propionibacterium acnes*. The intense use of antibiotics has led to the emergence of antibiotic-resistant *P. acnes* strains, which could transfer their antibiotic resistance genes to other commensal bacteria of the human skin microbiome through horizontal gene transfer [24,25,26,27,28]. A promising way to tackle the rising rates of antibiotic-resistant *P. acnes* strains is the use of endolysins [7,15,29,30].

The difficulty of the endolysins in attacking Gram-negative bacteria due to the outer membrane that prevents access to the peptidoglycan has already been overcome using different approaches. For example, modified forms of endolysins have been designed that have been fused with destabilizing peptide sequences [31,32,33]. On the other hand, some wild-type endolysins are able to interact with the surface of Gram-negative bacteria. Such inherent ability of some endolysins is connected with the presence of antimicrobial peptide (AMP)-like regions, which are, on their own, unexploited regions for engineering purposes and a significant source for novel antimicrobials.

In the present study, a putative endolysin (*Pa*Ami1), derived from *Propionibacterium acnes* phage PAC1, was characterized. Bioinformatics analysis of *Pa*Ami1 revealed a conserved N-acetylmuramoyl-L-alanine type-2 amidase (NALAA-2) catalytic domain. Type-2 amidases are zinc-dependent enzymes that catalyze the cleavage of the amide bond between N-acetylmuramoyl and L-amino acids in the PG layer of host cells [19]. The lytic and antimicrobial activities of *Pa*Ami1 were studied and characterized. Two engineered variants of the enzyme were designed as N-terminal fusions with antimicrobial peptides (AMP), and their lytic activity and thermostability were investigated and compared with those of the wild-type enzyme.

## 2. Results and Discussion

### 2.1. In Silico Characterization of PaAmi1

Endolysins represent an exciting approach with the potential to offer a potent and safe treatment for acne with minimal side effects and a low risk of inducing drug resistance. Considering that these possibilities carry important therapeutic implications in the management of acne, the present work was undertaken in order to characterize and evaluate a new endolysin from a *Propionibacterium acnes* phage.

A putative endolysin (*Pa*Ami1) in the genome of *Propionibacterium acnes* phage PAC1 (NCBI Accession Number: NC_028967.1) was identified (Accession Number: YP_009214894.1) and selected for further study. The PAC1 bacteriophage has been isolated from human skin microflora [34] and displays high lytic capacity towards cutaneous *P. acnes* strains. All known *P. acnes* phage genomes have a length of about 29 kbp and encode for 45–47 proteins. Among them, a single ORF encodes for an endolysin gene that is found in all genomes [33].

The endolysin gene from phage PAC1 (PAC1_20) is located at 15,505–16,362 bp in its genome. *Pa*Ami1 is composed of 285 amino acids with a theoretical molecular mass of 31,883 Da and an isoelectric point of 8.7. A BLASTp search using as a query the *Pa*Ami1 sequence showed that endolysins from different *P. acnes* bacteriophages are highly conserved, having >85% amino acid sequence identity. This high sequence homology between different endolysins from *P. acnes* phage genomes has also been observed by Liu et al. [35]. The authors sequenced forty-eight *P. acnes* bacteriophages from human skin follicles and found an 85–100% sequence identity between different strains and broad activity against seventy-four *P. acnes* skin isolates. The observed limited genetic diversity is probably a consequence of the bottleneck hypothesis or the evolutionary constraints imposed on phages and bacteria to maintain a single phage, thus limiting the spreading of phage resistance [35]. Another hypothesis for the restricted diversity of *P. acnes* bacteriophages may come from the niche in which they live, since *P. acnes* constitutes about 90% of the microbiota of the pilosebaceous unit [36].

**Figure 1:**
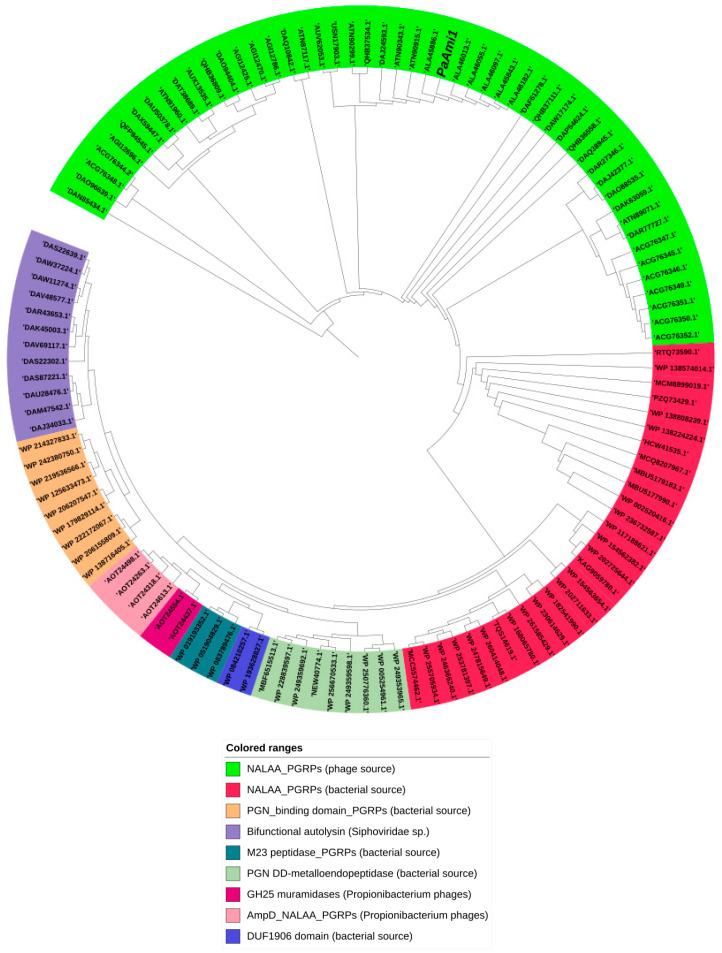
Phylogenetic analysis of *Pa*Ami1. Sequence alignments were performed using ClustalO [40] and analyzed using the Interactive Tree of Life (iTOL) v6 [41]. The phylogenetic tree is composed of nine different groups. The *Pa*Ami1 is clustered in the green group and is marked with italics. The majority of sequences in this group are annotated as N-acetylmuramoyl-L-alanine amidases and are derived from bacteriophages that infect Propionibacterium spp. Abbreviations: PGN, peptidoglycan; PGRPs, peptidoglycan recognition proteins; M23 peptidase_PGRPs, the M23 metallopeptidase family, which is also known as beta-lytic metallopeptidase; PGN DD-metalloendopeptidase,peptidoglycan DD-metallo endopeptidase family protein; GH25 muramidases, family of endo-N-acetylmuramidases that contain a glycosyl hydrolase family 25 (GH25) catalytic domain; AmpD_NALAA_PGRPs, AmpD amidases that belong to the NALAA family and PGRPs; DUF1906 domain, includes proteins that contain a domain of unknown function that is found in a set of uncharacterized hypothetical bacterial proteins.

Conserved Domains Database (CDD, https://www.ncbi.nlm.nih.gov/cdd/, accessed on 29 March 2022) analysis of *Pa*Ami1 sequence and search in the InterPro (https://www.ebi.ac.uk/interpro/search/sequence/, accessed on 29 March 2022) indicated that *Pa*Ami1 belongs to the peptidoglycan recognition proteins (PGRPs) superfamily (cl02712) and possessed a type-2 N-acetylmuramoyl-L-alanine amidase (NALAA-2, EC 3.5.1.28) domain (IPR002502) [37,38,39]. The conserved NALAA-2 catalytic domain (pfam01510) is located between 66 and 171 in the sequence. NALAA-2 are zinc-dependent enzymes that catalyze the hydrolytic cleavage of the amide bond between N-acetylmuramoyl and L-amino acids in the bacterial cell wall [19,37]. Phylogenetic analysis of *Pa*Ami1 (Figure 1) confirmed that it is grouped with N-acetylmuramoyl-L-alanine amidases from *Propionibacterium* phages. All *P. acnes* endolysins contain a highly conserved N-terminal domain associated with NALAA-2 activity, coupled to a 111-residue C-terminal extension whose function is unknown but likely mediates cell wall binding.

**Figure 2:**
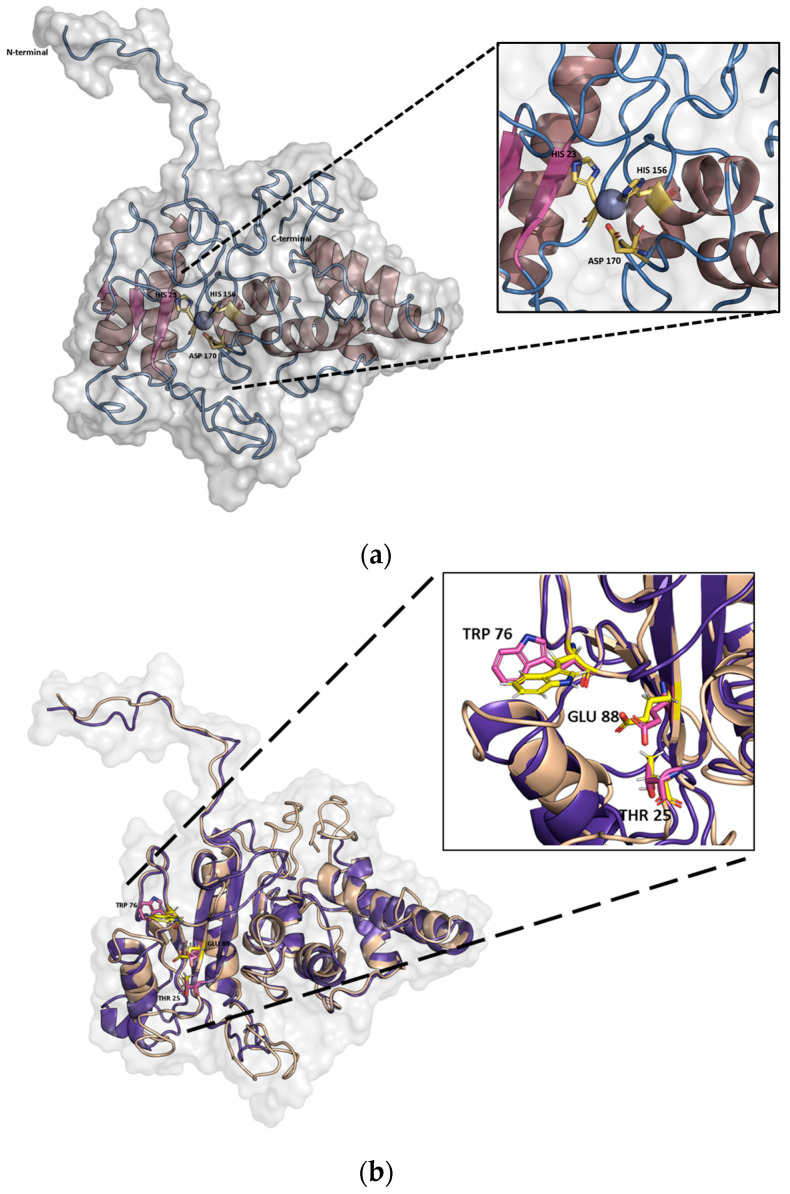

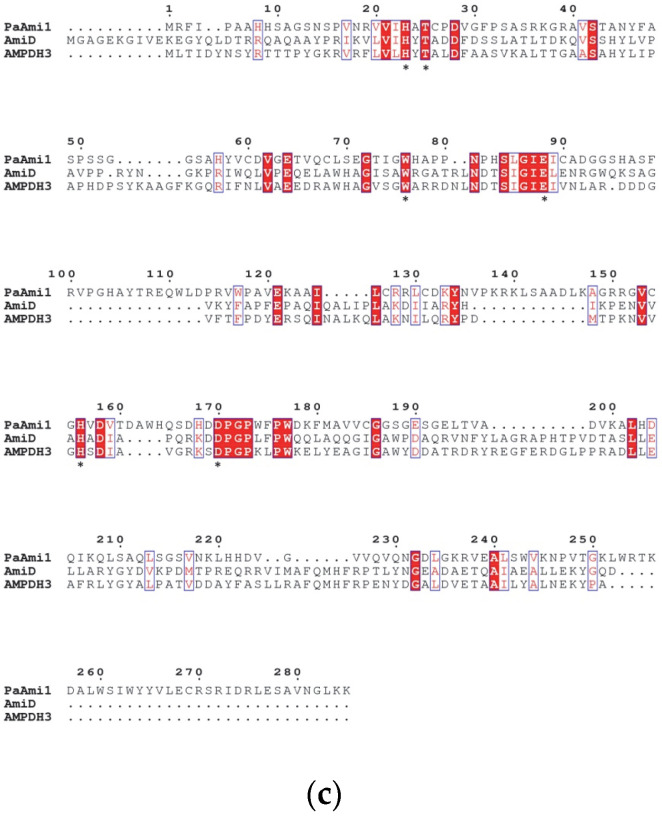
(**a**) Visualization of the predicted 3D structure of *Pa*Ami1. The zinc-binding residues (His23, His156, and Asp170) are shown in a stick representation and labeled; (**b**) Structural alignments of the predicted *Pa*Ami1 structure that is colored beige with that of the zinc-amidase from *Pseudomonas aeruginosa* AMPDH3 [47] colored purple. Important substrate-binding residues (Thr25, Trp76, and Glu88) are shown in a stick representation and labeled; (**c**) Sequence alignments of *Pa*Ami1 with two structurally homologous amidases (as identified by TM-align, [49]). AMPDH3: zinc-amidase from *Pseudomonas aeruginosa* (PDB accession number: 4BXD; [47]). AmiD: N-acetylmuramoyl-L-alanine amidase from *Escherichia coli* (PDB accession number 2WKX; [46]). Alignment was performed using ClustalO [40] and displayed using ESPript 3.0 [50]. Conserved areas are shaded. A column is framed if more than 70% of its residues are similar according to physicochemical properties. The conserved zinc-binding residues (His23, His156, and Asp170) and other important substrate-binding residues (Thr25, Trp76, and Glu88) are labeled with stars.

The 3D structure of *Pa*Ami1 was predicted using the I-TASSER server [42,43,44]. The most reliable model (C-SCORE −1.05) was used for structural analysis (Figure 2a). The predicted structure of *Pa*Ami1 adopts a fold similar to the structure of other NALAA-2 enzymes [45,46,47]. The catalytic zinc ion (Zn^2+^) is complexed with His23, His156, and Asp170. Ligand-binding site prediction using COFACTOR [43] and COACH [48] allowed the identification of Thr25, Trp76, and Glu88 as residues important for ligand-binding and catalytic activity (Figure 2b,c).

### 2.2. Expression and Purification of PaAmi1

The coding sequence of *Pa*Ami1 was cloned to the expression vector pETite T7 [51], which was used to transform a range of competent *E.coli* strains [*E.coli* BL21(DE3); BL21(DE3)pLysS; BL21Rosetta™(DE3); BL21Rosetta™(DE3)pLysS; BL21OverExpress™ C41(DE3); BL21 Shuffle^®^ T7 Express; BL21 Lemo21(DE3); BL21Origami™ (DE3); and BL21 Tuner™(DE3)] for selecting a strain capable of producing *Pa*Ami1 at high levels. The last two strains showed no growth after the transformation, so seven strains were selected for further studies. The enzyme was expressed as an insoluble protein in inclusion bodies (Supplementary Figure S1). Among all tested strains, BL21OverExpress™ C41(DE3) and BL21 SHuffle^®^ T7 Express displayed the highest expression level for *Pa*Ami1. The enzyme contains nine cysteine residues; therefore, the BL21 SHuffle^®^ T7 Express cells were selected as a suitable host, as typically this strain provides a suitable environment for Cys-rich proteins [52].

**Figure 3:**
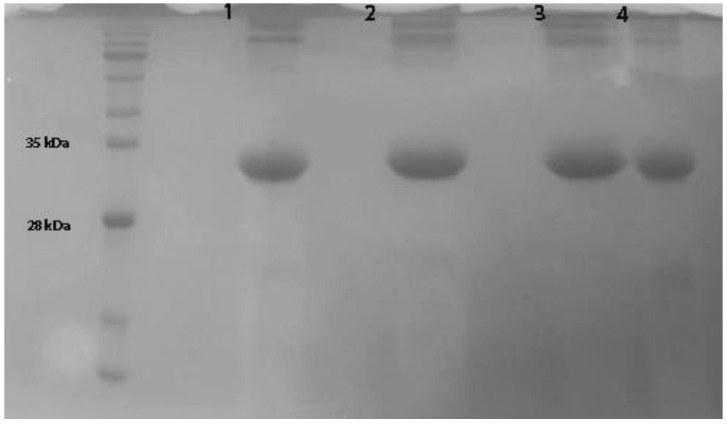
SDS-PAGE (12.5%) analysis of the purified *Pa*Ami1. Lane 1: *Pa*Ami1 in the solubilization buffer (2 M urea, 0.1 M Tris-NaOH, 1 mM DTT). Lanes 2–4: Solubilized *Pa*Ami1 was dialyzed against 20 mM Tris/HCl buffer, pH 9 (4 °C), on days 1, 3, and 7, respectively. Protein bands were stained with Coomassie Brilliant Blue R-250.

*Pa*Ami1 was solubilized and purified as denatured protein from inclusion bodies (Figure 3). Inclusion bodies were isolated by centrifugation, washed with non-ionic detergent, and solubilized under denatured conditions using urea (2 M urea, 0.1 M Tris/NaOH, 1 mM DTT, pH 12). The protein was slowly refolded using dialysis in 20 mM Tris/HCl buffer, pH 9, and stored at 4 °C until further use.

### 2.3. Demonstration of Lytic Activity and Substrate Specificity of PaAmi1

**Figure 4:**
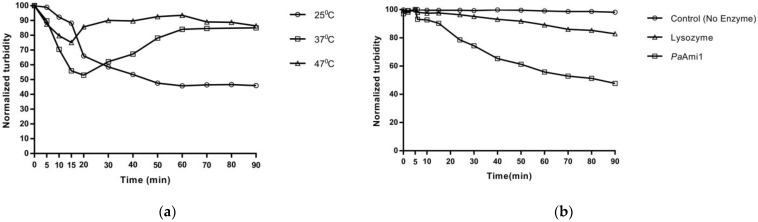

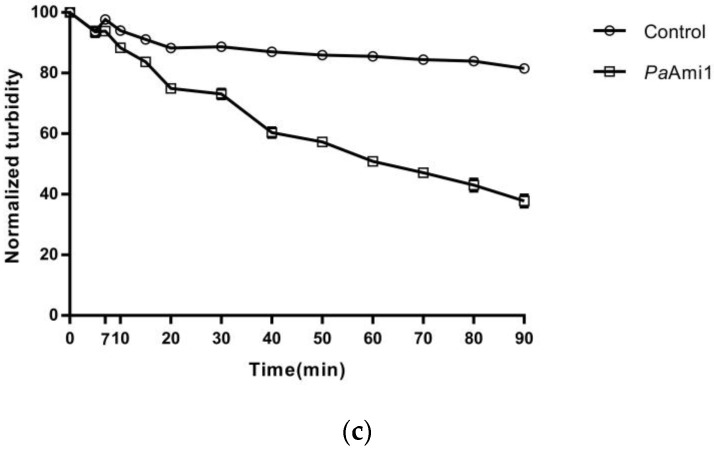
Demonstration of the lytic activity of *Pa*Ami1 using turbidity reduction assays. The following assays were performed in triplicate. (**a**) The turbidity reduction at 600 nm using *M. lysodeikticus* cell suspension. Absorbance was measured at specific time intervals for 90 min at three different temperatures (25 °C, 37 °C, and 47 °C). In all three cases, the reduction of the turbidity of a control assay (without *Pa*Ami1) was subtracted. The % lytic activity was calculated by taking the initial absorbance (600 nm) of the untreated sample (without *Pa*Ami1) as 100%; (**b**) Turbidity reduction assays of egg white lysozyme and *Pa*Ami1 using *Propionibacterium acnes* cells, previously stained with Remazol Brilliant Blue R dye. In both cases, assays were performed using 100 μg of enzymes (egg white lysozyme or *Pa*Ami1), and measurements were taken at 595 nm. In all cases, the % of lytic activity was calculated by taking as 100% the initial absorbance (595 nm) of each tested cuvette (control, egg white lysozyme, *Pa*Ami1); (**c**) Turbidity reduction assay of *Pa*Ami1 at 540 nm using purified *Propionibacterium acnes* peptidoglycan. All assays were performed for 90 min. The % of lytic activity was calculated by taking the initial absorbance (540 nm) of each tested cuvette (control and *Pa*Ami1) as 100%.

The lytic activity of the enzyme was initially assessed employing turbidity reduction assays, using as substrate the Gram-positive bacterium *M. lysodeikticus*. This assay was used as a reference method for assessing lytic activity [53]. The results (Figure 4a) showed that *Pa*Ami1 displayed high bacteriolytic activity towards *M. lysodeikticus* cells at 25 °C. The activity is strongly dependent on the incubation temperature. The enzyme appears to lose its activity after prolonged incubation (>15 min) at 37 °C and 47 °C (Figure 4a), indicating that thermal denaturation takes place.

The lytic activity of *Pa*Ami1 was also demonstrated and compared with that of egg white lysozyme by employing a substrate suspension of Remazol Brilliant Blue-R-labeled *Propionibacterium acnes* cells (Figure 4b). The results showed that at equivalent concentrations, *Pa*Ami1 displays higher lytic activity compared to that of egg white lysozyme. For example, at a 90 min assay, the turbidity of cell suspension was decreased by 50.42% using *Pa*Ami1, whereas only 15.26% by lysozyme (Figure 4b).

Moreover, the activity was assessed using purified PG instead of cell suspension. Figure 4c shows the turbidity assay of *Pa*Ami1using as substrate PG isolated from *Propionibacterium acnes* cells. *Pa*Ami1 was able to reduce the turbidity by 43.7%, confirming that *Pa*Ami1 catalyzes the lysis of the peptidoglycan layer of the host bacterial cell.

**Figure 5:**
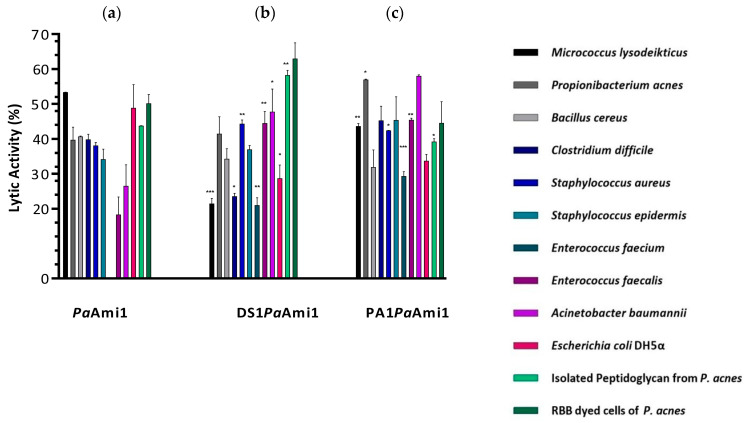
(**a**) Lytic activity of *Pa*Ami1 using turbidity reduction assays (ABS at 600 nm) under optimal conditions at 25 °C for 90 min. The study involved eight Gram-positive and two Gram-negative bacterial strains. Lytic activity was also demonstrated using isolated peptidoglycan from *P. acnes* cells or Remazol Brilliant Blue R-dyed dead *P. acnes* cells. In the case of *Pa*Ami1, the missing bar that corresponds to *E. faecium* cells indicates the absence of detectable activity towards this strain. Error bars represent the estimated standard deviation. The % lytic activity was calculated using Equation (1); (**b**,**c**) Lytic activity of the engineered variants (DS1*Pa*Ami1 and PA1*Pa*Ami1) using turbidity reduction assays (600nm) under optimal conditions at 25 °C for 90 min. Error bars represent the estimated standard deviation. The % of lytic activity was calculated using Equation (1). Bars represent the means and standard deviations of independent experiments. Statistical analysis of the (%) lytic activity of the enzymes was performed by one-way ANOVA using Dunnett’s test (GraphPad Prism 7.00 software). Bars with an asterisk are statistically different from the control bars (lytic activity of *Pa*Ami1). * Represent statistical significance with *p*-value ≤ 0.05; ** Represent statistical significance with *p*-value ≤ 0.01; *** Represent statistical significance with *p*-value ≤ 0.001.

The specificity of *Pa*Ami1 was examined by assessing its lytic activity against eight Gram-positive and two Gram-negative strains. The results are summarized in Figure 5. *Pa*Ami1 exhibited a broad range of activity across different microbial strains. The enzyme exhibits significantly higher lytic activity against *Streptococcus pyogenes* and *Bacillus cereus* compared to other species. High lytic activity was also observed towards *Propionibacterium acnes*, the native host of the bacteriophage PAC1 [54].

### 2.4. The Effect of pH and Divalent Metal Ions on PaAmi1 Activity

**Figure 6:**
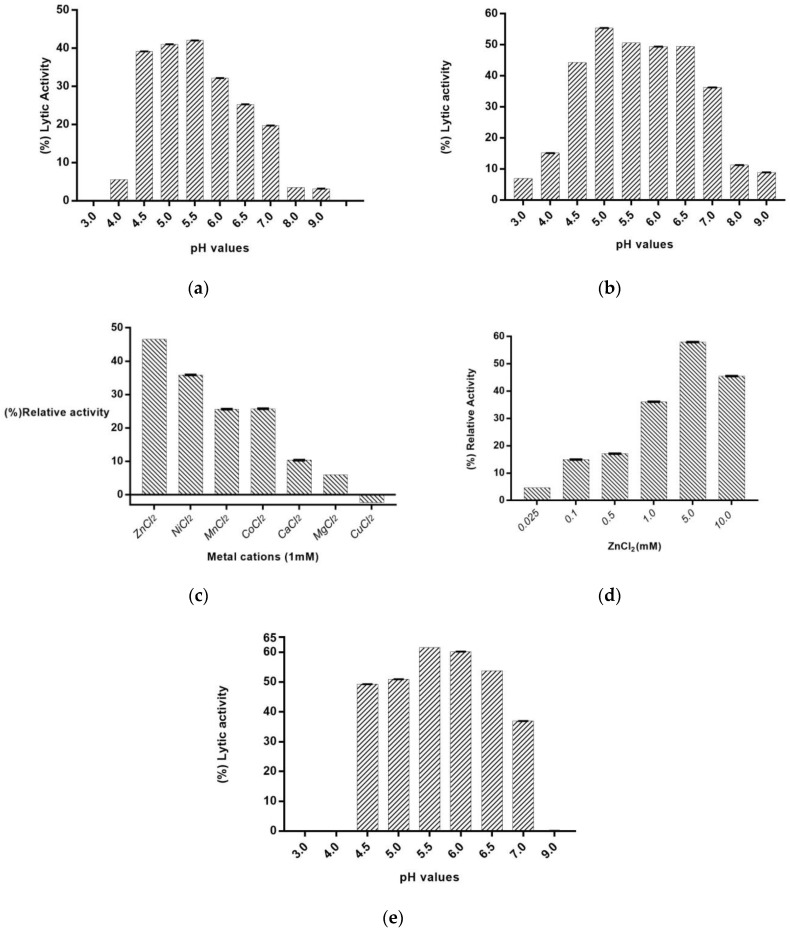
Effect of pH on the lytic activity of *Pa*Ami1. Enzyme activity was measured at 25 °C for 90 min using the turbidity reduction assay (600 nm). (**a**) Assays were performed using *M. lysodeikticus* cells as substrates in the absence of ZnCl_2_. The (%) lytic activity was calculated using Equation (1); (**b**) Assays were performed using *P. acnes* cells as substrates in the absence of ZnCl_2_. The % lytic activity was calculated using Equation (1); (**c**) Lytic activity of *Pa*Ami1 in the presence of different divalent metal ions (1 mM). Relative activities (%) are defined as a percentage of the lytic activity of 100 μg *Pa*Ami1 toward *M. lysodeikticus* cells in MES/NaOH, pH 6; (**d**) Relative activities of *Pa*Ami1 in the presence of different concentrations of ZnCl_2_ using as substrate *M. lysodeikticus* cells in 50 mM MES/NaOH, pH 6.0; (**e**) Effect of pH on lytic activity of *Pa*Ami1 using *M. lysodeikticus* cells in the presence of 5 mM ZnCl_2_. The (%) lytic activity was calculated using Equation (1). In all cases, error bars represent the standard error of the mean (SEM).

To determine the conditions required for optimal enzyme activity, the effects of pH and divalent metal cations were investigated (Figure 6). *Pa*Ami1 shows high lytic activity at pH values between 4.5 and 7.0 (Figure 6a,b). The optimum pH appears to be slightly dependent on the particular strain used as substrate (*M. lysodeikticus* vs. *P. acnes*) (for comparison, see Figure 6a,b). Using *P. acnes*, the enzyme displayed activity over a broader pH range compared to that displayed using *M. lysodeikticus* cells.

Considering that NALAA-2 are metal-dependent enzymes, the effect of a range of divalent metal ions (Zn^2+^, Ni^2+^, Mn^2+^, Mg^2+^, Co^2+^, Ca^2+^, and Cu^2+^) on lytic activity was evaluated (Figure 6c). Among all cations, Zn^2+^ appears to enhance significant (approx. 50%) lytic activity, confirming that *Pa*Ami1 displays zinc-dependent catalytic activity, similar to other amidases [19]. Furthermore, Ni^2+^ increases lytic activity by 36%. A smaller effect on enzyme activity was observed by Mn^2+^, Mg^2+^, Co^2+^, and Ca^2+^. On the other hand, the enzyme activity was inhibited in the presence of Cu^2+^, presumably due to the ability of Cu^2+^ to cause the oxidation of Cys residues [55].

The addition of 5 mM ZnCl_2_ (Figure 6d) showed the highest effect (57.9% increase) on the enzyme’s lytic activity. The effect of 5 mM Zn^2+^ on the optimum pH was also evaluated (Figure 6e). The results showed a slight effect of 5 mM Zn^2+^ on the pH-activity profile. It appears that the metal cation mainly affects the enzyme activity at pHs between 6 and 7.

### 2.5. Evaluation of the Inhibitory and Bactericidal Activity of PaAmi1 against Live Cultures of P. acnes

**Figure 7:**
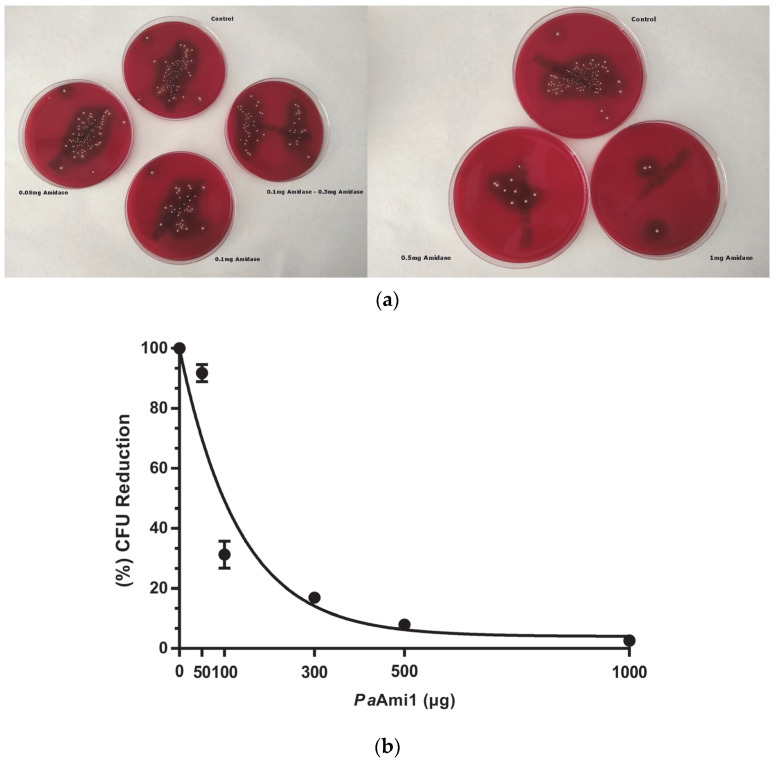
(**a**) Evaluation of different concentrations of *Pa*Ami1 (0.05–1.0 mg) on *P. acnes* growth. The control plate corresponds to growth in the absence of *Pa*Ami1. (**b**) Graphic demonstration of the number of colonies (CFU) of *P. acnes* cells that were formed on COL S+ plates after 3.5 h of incubation of *P. acnes* with different concentrations of *Pa*Ami1 under optimal conditions. Plates were incubated at 37 °C for 8 days under anaerobic conditions, and the colonies were counted and the data normalized. The number of colonies that were grown on the control plate was taken as 100%. Normalized data were analyzed using GraphPad Prism 7.00. Error bars represent standard deviations (± SDs).

The effect of different concentrations of purified *Pa*Ami1 on live cultures of *P. acnes* was evaluated. Figure 7 shows a dose-response effect of *Pa*Ami1 (50–1000 μg) on *P. acnes* growth. It is observed that the number of colonies significantly decreased as the quantity of *Pa*Ami1 increased. Treatment with 50 μg of *Pa*Ami1 had little effect on the growth of *P. acnes* cells, whereas using 1000 μg of *Pa*Ami1 caused a dramatic effect.

### 2.6. ‘Artilysation’ of the Endolysin PaAmi1

A protein engineering strategy was undertaken, aiming to alter the enzyme’s specificity towards bacterial pathogens. The approach, so-called ‘artilysation’, was based on the fusion of short antimicrobial peptides with membrane penetrating activity to the enzyme’s N-terminus [31,32,33,34,35]. The engineering strategy aimed to combine the lytic activity of *Pa*Ami1 with the membrane-penetrating activity of antimicrobial peptides.

Two different AMPs were fused at the N-terminus of *Pa*Ami1. The first peptide, designated DS1, has the following amino sequence: RIRLLQRFNKR. DS1 was selected following searches in the DBAASP v3.0 database (Collection of Antimicrobial Peptides, [56]). DS1 has been experimentally validated to exhibit antimicrobial activity against *Propionibacterium* spp. and was selected based on its low IC50 and MIC values [57]. The second AMP, designated as PA1, was selected by searching the genomes of five *Propionibacterium* phages: PAC1 (NCBI Accession Number: NC_028967.1); QueenBey (NCBI Accession Number: NC_031005.2); G4, (NCBI Accession Number: NC_041895.1); B5, (NCBI Accession Number: NC_003460.1); and P2, (NCBI Accession Number: KY926793.1). With this approach, we evaluated the hypothesis that encrypted AMP-like regions exist in the genome of bacteriophages that can be exploited to design and develop novel engineering artilysins. We bioinformatically analyzed (Supplementary Figure S2) the genome of the five phages for potential AMP-like sequences using the MACREL algorithm [58]. A subset of 48 putative AMPs was predicted from the screening and further assessed using the CAMPR3 algorithm [59,60] for selecting shorter antimicrobial regions within the peptides. We focused on short peptides (e.g., less than <11 amino acids in length) for easy fusion with *Pa*Ami1 without interfering with its catalytic activity. The derived short AMPs were assessed and ranked using the deep learning/physicochemical property-based Al4AMP algorithm and further validated employing the DBAASP v3.0 and AxPEP algorithms [59,61,62,63,64]. A comparative analysis of the predicted AMPs from the genome of Propionibacterium phages is shown in Supplementary Table S1. A candidate AMP with the amino sequence RVFRRAARIAQ (PA1) was selected for further study. The PA1 sequence is derived from the PAC1 genome, and based on all algorithms used (Al4AMP, DBAASP v3.0, and AxPEP), it was collectively predicted to display strong antimicrobial activity. The toxicity of the two selected AMPs (DS1 and PA1) towards mammals was examined using the HAPPENN algorithm [65], where the hemolytic activity of DS1 and PA1 was assessed and found to be negligible.

The selected AMPs (DS1 and PA1) were fused at the N-terminus of *Pa*Ami1, and the engineered enzymes, DS1*Pa*Ami1 and PA1*Pa*Ami1, were expressed and purified as described for the wild-type enzyme. The lytic activity of the three enzymes was assessed against eight Gram-positive and two Gram-negative strains. In addition, their lytic activity was tested using isolated peptidoglycan from *P. acnes* cells or Remazol Brilliant Blue R-dyed *P. acnes* cells. The results showed (Figure 5b,c) that the engineered variants exhibited altered lytic activity and specificity towards the tested strains compared to the wild-type enzyme. PA1*Pa*Ami1 and DS1*Pa*Ami1 showed higher lytic activity towards *P. acnes* cells, which reached 56.9 ± 0.2% and 41.4 ± 3.5%, respectively, compared to the wild-type (39.7 ± 2.6%). In addition, both engineered variants exhibited improved lytic activity towards the Gram-negative *Acinetobacter baumannii*, which is considered today to be the most dangerous multidrug-resistant pathogen [66]. Similarly, improved activity was observed against the most common species of Enterococci, *Enterococcus faecalis* and *Enterococcus faecium,* which are the leading causes of enterococcal infections [67]. Noteworthy, the wild-type enzyme did not display lytic activity towards the *E. faecium* cells, whereas significant activity was found for both engineered variants and especially for PA1*Pa*Ami1. This is an important outcome of the study since it provides clear evidence that mining AMPs from the bacteriophage genomes may represent an efficient and rapid approach for expanding the available antimicrobial resources. All these results point to the conclusion that our engineered strategy and the selected AMPs, fused at the N-terminus of *Pa*Ami1, are an effective approach for engineering endolysins with altered or improved lytic activity. A relevant strategy has been adopted by Vázquez et al., 2021 [68]. They bioinformatically analyzed the C-terminal end of a collection of lysins from phages infecting the Gram-negative genus Pseudomonas. They selected and tested two putative membrane-interacting endolysins and found that they were active against *Pseudomonas aeruginosa* and other Gram-negative bacterial pathogens.

### 2.7. Thermostability of PaAmi1, DS1PaAmi1, and PA1PaAmi1

**Figure 8:**
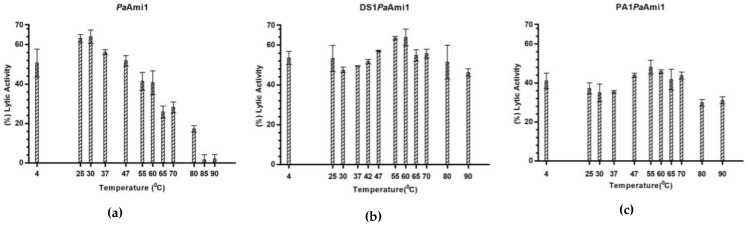
The effect of temperature on the lytic activity of the wild-type enzyme *Pa*Ami1 on *M. lysodeikticus* cells (**a**) and the engineered variants DS1*Pa*Ami1 on *P. acnes* cells (**b**) and PA1*Pa*Ami1 on *M. lysodeikticus* cells (**c**). The thermostability was evaluated by incubating the enzymes for 10 min at different temperatures (4–90 °C). Absorbance measurements were taken at 600 nm, and the lytic activity was calculated using Equation (1). In all cases, error bars represent the means ± SDs of independent experiments.

The effect of temperature on the operational stability of the wild-type and engineered variants was investigated by measuring the remaining enzyme activity after incubation (10 min) of the enzymes at different temperatures. The results (Figure 8) revealed that the activity of the wild-type enzyme was not affected at temperatures <30 °C; however, its activity rapidly declined at higher incubation temperatures. On the other hand, the engineered variants DS1*Pa*Ami1 and PA1*Pa*Ami1 exhibited much higher thermostability compared to the wild-type enzyme, as they showed catalytic activity even after incubation at 90 °C. This unexpected finding is presumable due to the presence of the AMP sequences. Both DS1 and PA1 sequences are positively charged; they possess four Arg residues, which may stabilize the N-terminal domain of the enzyme. The contribution of Arg residues to protein thermostability has been well established in several thermostable proteins [69]. A similar stabilization effect has been observed with other artilysins. For example, ‘artilysation’ of endolysin λSa2lys improves its structural stability [55].

## 3. Materials and Methods

### 3.1. Biocomputing Analysis

*Pa*Ami1 homologue sequences were sought using BLASTp (Basic Local Alignment Search Tool) [70]. The resulting sequences were aligned using ClustalOmega [40,71]. The phylogenetic tree of *Pa*Ami1 that was generated by ClustalOmega was analyzed and portrayed using the online tool iTOL [41]. The 3D structure of *Pa*Ami1 was predicted using the I-TASSER server [42,43,44]. I-TASSER generated five models. The quality of the models was assessed using C-SCORE [42,43,44].

Biocomputing analysis for the selection of antimicrobial peptides was achieved by searching the DBAASP v3.0 database [56] and the CAMPR3 database (Collection of Antimicrobial Peptides, [59,60]). In addition, search of the genomes of five *Propionibacterium* phages [PAC1 (NCBI Accession Number: NC_028967.1), QueenBey (NCBI Accession Number: NC_031005.2), G4, (NCBI Accession Number: NC_041895.1), B5 (NCBI Accession Number:NC_003460.1), and P2 (NCBI Accession Number: KY926793.1)] for potential AMP sequences was achieved using the MACREL algorithm [58] and further analyzed using a range of antimicrobial prediction tools and algorithms: CAMPR3, DBAASP v3.0, AI4AMP and AxPEP (AmPEP and RF-AmPEP, 30) and APD3 [56,59,60,61,62,63,64,72,73,74]. Sequences were ranked according to AI4AMP scores, ranging from 0 to 1, with a threshold of 0.5 [62]. Sequences with scores <0.5 were discarded. The toxicity and hemolytic activity of the selected AMPs towards mammals were examined using the HAPPENN tool [65]. All databases were accessed on 14–15 March 2022.

### 3.2. Synthesis and Cloning of PaAmi1

The *Pa*Ami1 gene was codon-optimized for *E. coli* expression and synthesized (Eurofins Genomics, Abersberg, Germany). Amplification of *pa*Ami1gene was performed by PCR using the In-Fusion^®^ HD Cloning Kit (Takara Bio USA Inc, Mountain View, CA, USA) and the following primers: *PaAmi*1F (5′-GAA GGA GAT ATA CAT ATG CGC TTC ATT CCG GCT-3′) and *Pa*Ami1R (5′-GTG ATG GTG GTG ATG ATG CTT CTT CAG ACC ATT GAC CGC-3′). The PCR reaction was carried out in a total volume of 20 μL, containing the following: 10 μL Clone Amp HiFi PCR Premix, 0.5 μM forward and reverse primers, 20 ng template DNA, and 6 μL H_2_O. The conditions used in the thermocycler were an initial denaturation at 98 °C for 4 min. The PCR protocol comprised 35 cycles of 10 s at 98 °C, 15 s at 55 °C (annealing temperature), and 15 s at 72 °C (extension temperature). A final extension time of 10 min at 72 °C was performed after the 35th cycle. The PCR product was cloned into a T7 expression vector (pETite C-His Kan vector, Lucigen, Middleton, WI, USA) [51], which possessed a C-terminal hexahistidine (6-His) tag sequence. The resulting expression construct was sequenced and transformed into nine different competent *E. coli* expression strains [BL21(DE3), BL21(DE3)pLysS, BL21Rosetta™(DE3), BL21Rosetta™(DE3)pLysS, BL21OverExpress™ C41(DE3), BL21 Shuffle^®^ T7 Express, BL21 Lemo21(DE3), BL21Origami™ B(DE3), and BL21 Tuner™(DE3)] in order to determine the best expression strain for *Pa*Ami1.

### 3.3. Expression and Purification of PaAmi1

Overnight culture of *E. coli* BL21 Shuffle^®^ T7 Express cells in LB medium containing ampicillin (100 μg/mL) was used to inoculate fresh 2xYT medium (ampicillin 100 μg/mL) and was incubated at 37 °C under shaking at 180rpm until OD at 600 nm reached 0.5 to 0.6 [52]. Furthermore, the expression of *Pa*Ami1 was induced by the addition of 0.5 mM IPTG followed by incubation at 20 °C for 20 h at 160 rpm. Bacterial cells were suspended in Lysis buffer (50 mM sodium phosphate, 300 mM sodium chloride, 10 mM imidazole, pH 8.0) and lysed under sonication. The suspension was centrifuged at 13,000× *g* for 7 min, and the supernatant was discarded since *Pa*Ami1 was expressed as an insoluble protein in inclusion bodies (IBs). The pellet was washed three times with wash buffer [2% (*v*/*v*) Triton X-100] and two times with double-distilled water. Each time, the pellet was vortexed and centrifuged at 13,000× *g* for 15 min. Next, the pellet was solubilized with the following buffer: 2 M urea-0.1 M Tris-NaOH buffer, pH 12, containing 1 mM DDT. Protein refolding was carried out with overnight dialysis against 20 mM Tris, pH 9. The enzyme solution was stored at 4 °C until further use [75,76]. Protein purity was confirmed by sodium dodecyl sulfate polyacrylamide gel electrophoresis (SDS-PAGE, 12.5% separating gel; 6% stacking gel).

### 3.4. Lytic Activity Assays

Bacterial cells were suspended in appropriate buffer to a final OD_600nm_ of 0.5–0.6, and the reduction of the turbidity of the cell suspension was monitored for 90 min at 25 °C in the presence of *Pa*Ami1 (100 μg/mL, unless stated otherwise). The course of the reaction was monitored by taking the absorbance at specific time intervals. Likewise, in the control cuvette, the same components were added, but instead of *Pa*Ami1, dialysis buffer was added. The lytic activity (%) was calculated after a 90 min reaction using Equation (1):

(1) $$\left[\frac{\left[{\Delta \mathrm{OD}}_{0\text{–}90\min}\left(\mathrm{test}\right)\right]}{\mathrm{initial}\;\mathrm{absorbance}\;\left(\mathrm{test}\right)}\text{×}100]-[\frac{{(\Delta \mathrm{OD}}_{0\text{–}90\min}\left(\mathrm{control}\right)}{\mathrm{initial}\;\mathrm{absorbance}\;\left(\mathrm{control}\right)}\text{×}100\right]$$

In that case, the reduction of the control assay (without enzyme) is subtracted, and the effect of the enzyme on the cell suspension is evident. The antimicrobial spectrum of *Pa*Ami1 was assessed by measuring the turbidity reduction of different bacterial cell suspensions *(Enterococcus faecalis, Bacillus cereus, Micrococcus lysodeikticus, Clostridium difficile, Streptococcus pyogenes, Staphylococcus epidermis, Staphylococcus aureus, Propionibacterium acnes, Enterococcus faecium,* and *Acinetobacter baumannii*) at 600 nm using 50 mM KH_2_PO_4_/K_2_HPO_4_, pH 6.24. The lytic activity of *Pa*Ami1 was evaluated using Equation (1). Lyophilized *Micrococcus lysodeikticus* cells were obtained from Sigma-Aldrich (Sigma-Aldrich Co., St. Louis, MO, USA). DH5a *E. coli* was obtained by Thermo Fisher Scientific, Waltham, MA, USA. The other bacterial strains were much a appreciated gift from the Department of Microbiology, “Aghia Sophia” Children’s Hospital, Athens (Dr. A. Stathi) and described elsewhere [3]. The bacterial strains were autoclaved before use. Autoclaved bacteria were washed once with the reaction buffer (50 mM KH_2_PO_4_/K_2_HPO_4_, pH 6.24) before being used in the turbidity reduction assay.

### 3.5. Effect of pH, Divalent Metal Ions, and Temperature

The effect of pH (pH 3–9) on the activity of *Pa*Ami1 was assessed using the turbidity reduction assay and employing *Micrococcus lysodeikticus* or *Propionibacterium acnes* cells as substrate. The following buffers (50 mM) were used: Glycine/HCl pH 3.0; CH_3_COOH/CH_3_COONa pH 4.0; CH_3_COOH/CH_3_COONa pH 4.5; CH_3_COOH/CH_3_COONa pH 5.0; MES/NaOH pH 5.5; MES/NaOH pH 6.0; MES/NaOH pH 6.5; HEPES, pH 7.0; HEPES pH 7.5; HEPES pH 8.0; and Na_2_B_4_O_7_/H_3_BO_3_ pH 9. The lytic activity of *Pa*Ami1 was also evaluated in the presence of different divalent metal ions (1 mM): ZnCl_2_, NiCl_2_, MnCl_2_, MgCl_2_, CoCl_2_, CaCl_2_, and CuCl_2_ or 5 mM ZnCl_2_. The activity of *Pa*Ami1 was performed in three temperatures (25 °C, 37 °C, and 47 °C) in order to determine the optimal temperature for lytic activity.

### 3.6. Turbidity Reduction Assays Using Isolated Peptidoglycan

Peptidoglycan from *P. acnes* cells was isolated according to Fukushima and Sekiguchi, (2016) [77]. Following purification, the isolated peptidoglycan was washed five times with Milli-Q water, lyophilized, and stored at −20 °C. Turbidity reduction assays using isolated peptidoglycan were performed as described above (using cell suspension), with the only exception that the absorbance of the peptidoglycan suspension was measured at 540 nm.

### 3.7. Turbidity Reduction Assays Using Remazol Brilliant Blue R-Dyed Cells

Autoclaved *Propionibacterium acnes* cells were dyed with Remazol Brilliant Blue R (RBB-dyed cells) according to Zhou et al., 1988 [78]. The RBB-dyed cell suspension after being washed with double-distilled water, was lyophilized and stored at −20 °C. The lytic activity was measured as described above (using cell suspension), with the only exception that the absorbance of the RBB-dyed cells was monitored at 595 nm.

### 3.8. Lytic Activity of PaAmi1 Using Live Propionibacterium Acnes Cells on Agar Plates

*Propionibacterium acnes* cells were cultivated, and then a 0.5 McFarland standard was prepared. Appropriate dilutions were made until approximately 10^4^ cells were suspended in each reaction tube containing 50 mM MES/NaOH buffer, pH 6.0, and ZnCl_2_ (5 mM). Furthermore, different amounts of purified *Pa*Ami1 were added (0.05–1.0 mg), and the reactions were left at room temperature and under stirring for 3.5 h. A control reaction was also prepared under the same conditions, but instead of *Pa*Ami1, dialysis buffer (20 mM Tris, pH 9.0) was added. Following incubation, a sample (10 μL) of each reaction was transferred to an agar plate (COL S +) and incubated at 37 °C for 8 days under anaerobic conditions.

### 3.9. Fusion of PaAmi1 with Antimicrobial Peptides (AMP)

Fusion of the AMPs DS1 (RIRLLQRFNKR) and PA1 (RVFRRAARIAQ) at the N-terminus of *Pa*Ami1 to give the engineered variants DS1*Pa*Ami1 and PA1*Pa*Ami1 was achieved by PCR. The amplification of the whole expression vector pETite C-HisKan vector (Lucigen, Middleton, WI, USA) was performed by PCR using the KAPA Taq PCR Kit (KAPA Biostystems Pty, Cape Town, South Africa) and the following forward primers:

ds1F(5′-ATGCGCATTCGCTTATTACAGCGCTTCAACAAGCGCCGCTTCATTCCGGCT-3′) and
pa1F(5′-ATGCGCGTATTCCGCCGCGCAGCACGCATTGCACAGCGCTTCATTCCGGCT-3′).

As a reverse primer in both PCRs, the pETite reverse primer was used (5′-CTCAAGACCCGTTTAGAGGC- 3′). The PCR reaction was carried out in a total volume of 15 μL, containing the following: 7.5 μL Clone Amp HiFi PCR Premix, 0.2 μM forward and reverse primers, 2 ng template DNA, and 6.6 μL H_2_O. The conditions used in the thermocycler were an initial denaturation at 98 °C for 4 min. The PCR protocol comprised 30 cycles of 10 s at 98 °C, 15 s at 62 °C (annealing temperature), and 16 s at 72 °C (extension temperature). A final extension time of 10 min at 72 °C was performed after the 30th cycle. The amplified PCR products were incubated with T4 DNA ligase (Takara Bio USA Inc, Mountain View, CA, USA) at 16 °C for 16 h. Following ligation, the plasmids were treated with the restriction enzyme Dpn I (FastGene, Nippon Genetics, Tokyo, Japan), and the recombinant constructs were used to transform competent *E. coli* stellar cells for sequencing, and *E. coli* BL21 OverExpress™ C41(DE3) cells for heterologous expression.

### 3.10. Expression, Purification, and Lytic Activity of DS1PaAmi1 and PA1PaAmi1

Overnight culture of *E. coli* BL21 OverExpress™ C41(DE3) cells in an LB medium containing ampicillin (100 μg/mL) was used to inoculate fresh 2 × YT medium (ampicillin 100 μg/mL) and was incubated at 37 °C under shaking at 180 rpm until the OD at 600 nm reached 0.5 to 0.6. The expression of DS1*Pa*Ami1 and PA1*Pa*Ami1 was induced with 0.5 mM IPTG, followed by incubation at 20 °C for 20 h at 160 rpm. Purification of DS1*Pa*Ami1 and PA1*Pa*Ami1 was performed as described for the wild-type enzyme *Pa*Ami1. The bacteriolytic activity of DS1*Pa*Ami1 and PA1*Pa*Ami1 was achieved as described for the wild-type enzyme *Pa*Ami1.

### 3.11. Thermostability Analysis

The thermostability of the wild-type and the engineered variants (DS1*Pa*Ami1 and PA1*Pa*Ami1) was evaluated by incubating the enzymes (for 10 min) at different temperatures (4–90 °C) in a 50 mM MES/NaOH buffer at pH 6. Following incubation, the remaining enzyme activity was measured using turbidity reduction assays (bacterial cell suspensions). The activity of *Pa*Ami1 and PA1*Pa*Ami1 was measured using *M. lysodeikticus* cells as substrates, while the activity of DS1*Pa*Ami1 was measured using *P. acnes* cells. This is because DS1*Pa*Ami1 displayed low lytic activity on *M. lysodeikticus* cells.

## 4. Conclusions

Enzyme therapy utilizing endolysins is a promising solution to the rising problem of antibiotic-resistant bacteria. In the present work, a new NALAA-2 from *P. acnes* phage PAC1 was identified, expressed, and characterized. The lytic activity of *Pa*Ami1 was established using a range of bacterial pathogens as well as purified peptidoglycan from *P. acnes*. An engineering strategy based on the fusion of short antimicrobial peptides with their N-termini was employed, aiming at creating new variants with altered lytic activity and specificity. We evaluated the hypothesis that antimicrobial peptide (AMP)-like regions exist in the genome of bacteriophages that can be exploited to design novel artilysins. One AMP sequence that was in silico predicted from the genome of the PAC1 phage, when fused at the N-terminus of *Pa*Ami1, gave an engineered variant with enhanced operational stability and lytic activity towards *P. acnes* and the enterococci species *Enterococcus faecalis* and *Enterococcus faecium*. The engineered enzyme can be further studied as a potential antimicrobial agent with applications in the cosmetic and pharmaceutical industries.

## Data Availability

All data are included in the article.

## Supplementary Materials

The following supporting information can be downloaded at: https://www.mdpi.com/article/10.3390/ijms24108523/s1.

## Abbreviations

PG, Peptidoglycan; AMP, Antimicrobial peptide; AMR, Antimicrobial resistance; CDD, Conserved Domains Database; DSF, Differential Scanning Fluorimetry; DAPI, 4′,6-diamidino-2-phenylindole; EthD-III, Ethidium Homodimer III; MIC, minimal inhibition concentration; MDR, Multidrug resistant; NALAA, N-acetylmuramoyl-L-alanine amidase; IBs, Inclusion bodies; IPTG, Isopropyl-β-D-thiogalactopyranoside; SDS-PAGE,Sodium dodecyl sulfate polyacrylamide gel electrophoresis; LB, Luria and Bertani medium; 2 × Yeast Tryptone medium, 2 × YT medium; OD, Optical density; RBBR, Remazol Brilliant Blue-R; WHO, World Health Organization; ORF, Open Reading Frame.
