# Supplementary material for: Characterization and Engineering Studies of a New Endolysin from the Propionibacterium acnes Bacteriophage PAC1 for the Development of a Broad-Spectrum Artilysin with Altered Specificity
Source: Int J Mol Sci. 2023 May 10;24(10):8523. doi: 10.3390/ijms24108523 (PMC10218239; doi:10.3390/ijms24108523)
Supplement: Supplementary file 1 [file ijms-24-08523-s001.zip › ijms-2354400-supplementary.docx]

Supplementary materials

Supplementary Table S1. Summary of the properties of the predicted AMPs. Sequences are ranked from top to bottom according to the highest score calculated by AI4AMP [48]. Column 1: The sequence of the predicted AMPs. In red characters are shown the hydrophobic residues. Column 2: The estimated total hydrophobic residues (%) and the net charge of the peptides. Column 3: The antimicrobial score based on Al4AMP prediction. Scores are ranging from 0 to 1 with a threshold of 0.5 [49]. Column 4: Classification of sequences as antimicrobial (AMP) or non-antimicrobial (NAMP) using the DBAASP v3.0 [47]. Columns 5 and 6: Prediction of antimicrobial activity using the AxPEP. Two different algorithms (AmPEP, [51] and RF-AmPEP30, [50] were employed. Values 1 and 0 stand for antimicrobial and non-antimicrobial peptide, respectively. The #3 sequence (PA1) that displayed the highest AI4AMP score and confirmed antimicrobial activity by the three algorithms (DBAASP v3.0, AmPEP and RF-AmPEP30) was selected for further studies.

| **#** | **(1)**  **Sequence** | **(2)**  **Total hydrophobic residues (%)/total net charge** | **(3)**  **Score in AI4AMP** | **(4)**  **DBAASP v3.0** | **(5)**  **AmPEP** | **(6)**  **RF-AmPEP30** |
| --- | --- | --- | --- | --- | --- | --- |
| **1** | RIASILWRVGR | 55/ +3 | 0.9987 | AMP | 0 | 1 |
| **2** | PARVFRRAARI | 55/ +4 | 0.9953 | AMP | 0 | 1 |
| **3** | **RVFRRAARIAQ** | **55/ +4** | **0.9922** | **AMP** | **1** | **1** |
| **4** | IASILWRVGRG | 55/ +2 | 0.9839 | AMP | 0 | 1 |
| **5** | SILWRVGRGLA | 55/ +2 | 0.9836 | AMP | 0 | 1 |
| **6** | RIGTTYKGGKR | 9/ +4 | 0.9815 | NAMP | 0 | 1 |
| **8** | ARVFRRAARIA | 64/ +4 | 0.9647 | AMP | 1 | 1 |
| **8** | ASILWRVGRGL | 55/ +2 | 0.9595 | AMP | 0 | 1 |
| **9** | VPARVFRRAAR | 55/ +4 | 0.9231 | AMP | 0 | 1 |
| **10** | GVIVLAMGVRR | 64/ +2 | 0.8841 | NAMP | 0 | 1 |
| **11** | AVPARVFRRAA | 64/ +3 | 0.8647 | AMP | 0 | 1 |
| **12** | FRIASILWRVG | 64/ +2 | 0.8363 | NAMP | 1 | 1 |
| **13** | KEGTNRRRCLT | 18/ +3 | 0.7380 | AMP | 0 | 1 |
| **14** | TYRSKKRLRQL | 18/ +5 | 0.7249 | NAMP | 1 | 1 |
| **15** | PHKAALAIIAA | 73/ +1.25 | 0.7126 | NAMP | 0 | 1 |
| **16** | EGTNRRRCLTV | 27/ +2 | 0.6755 | NAMP | 0 | 0 |
| **17** | AQAVPARVFRR | 55/ +3 | 0.6480 | AMP | 0 | 0 |
| **18** | AATYRSKKRLR | 27/ +5 | 0.6412 | NAMP | 0 | 1 |
| **19** | ILARVRAGGGV | 55/ +2 | 0.5955 | NAMP | 1 | 1 |
| **20** | FTAIAFASVAC | 82/ 0 | 0.5353 | NAMP | 1 | 1 |
| **21** | RGLVTALGVGF | 55/ +1 | 0.5195 | AMP | 1 | 1 |
| **22** | TFRIASILWRV | 64/ +2 | 0.5024 | NAMP | 1 | 1 |

**
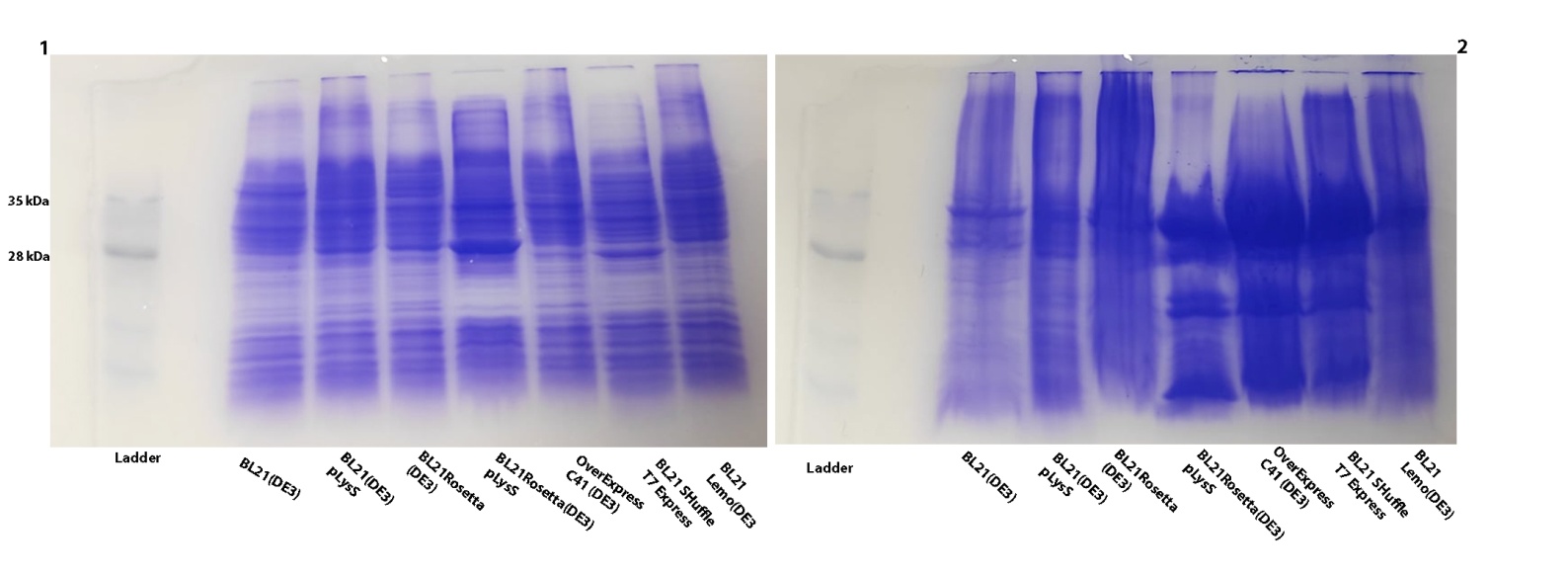
**

Supplementary Figure S1. Expression of *Pa*Ami1 in *E.coli* BL21(DE3) strains. Competent strains were transformed with the recombinant plasmid, grown in 2xYT medium at 20 ⁰C for 20 h, harvested, lysed by sonication and centrifuged. Both supernatant and pellet fractions were analysed by 12.5% SDS-PAGE. 1: SDS-PAGE analysis of the supernatants of the crudes. 2: SDS-PAGE analysis of the pellet.
